# Personal

**Published:** 1915-06

**Authors:** 


### PERSONAL.

Announcement of removal, travel, and other matters of interest are requested. Please report errors in the listing of any physician in the State and other directories, that we may co-operate with the State Society in securing a correct list.

---

Dr. A. L. Benedict of Buffalo would greatly appreciate information as to prehistoric Indian village and burial sites and especially prompt notice of any new discoveries.

---

Our Associate Editor, Dr. J. George Adami of Montreal has left for England, where he will serve on a commission to prepare a medical history of the war.

---

Our Associate Editor, Dr. Rene Gaultier of Paris, is Aide-Major of the first class, with the 65th Regiment of Infantry, headquarters at Nantes. As soon as the stress of his duties is lessened, he will send an article on the surgical and medical aspects of the campaign.

---

Dr. Charles W. Bethune of Buffalo, has moved his office to The Matthewson, 290 S. Elmwood Ave.

---

Dr. R. C. Miller has returned from Seattle and will locate at 596 Broadway, Buffalo.

---

Dr. Edward J. Ballou of Gardenville, has moved to Buffalo.

---

Dr. John Chalmers of Buffalo, is making the Canon-Coast-Yellowstone trip.

---

Dr. Victor M. Rice of Batavia has been appointed Health Officer, succeeding Dr. Cornelius F. McCarthy.

---

Dr. Otto V. Huffman, Secretary of the N. Y. State Board of Medical Examiners and Secretary and Treasurer of the Federation of State Medical Boards and editor of its publications, has been elected Secretary of the Faculty and Executive Officer of the L. I. College Hospital, succeeding the late Dr. Joseph H. Raymond.

---

The automobile of Dr. Earl P. Lothrop of Buffalo was stolen May 14 but was recovered the next day. It was apparently not damaged but had been driven quite a distance.

---

Dr. John M. Mesmer of Buffalo has moved his residence to 9 Linwood Place.

Dr. J. B. Croff of Buffalo has moved to 175 Franklin St.

---

Dr. Thomas Foley of Buffalo has located at 400 N. Main St., Elmira. Practice limited to eye, ear, nose and throat.

---

Dr. W. C. Dingman of Rochester has moved to 27 Faraday St.

---

Dr. W. B. Cochrane of Rochester has moved to 1335 Park Ave.

---

Drs. A. Walter Suiter and George Graves of Herkimer, having served as Secretary and Treasurer respectively of the County Society for over 40 years, were guests of honor at a banquet of the Medical Society of the County of Herkimer, April 21. A Blue Lodge pin was presented to the former and a Mystic Shriner pin to the latter.

---

Dr. William G. Taylor of Buffalo, returned from California, the middle of May.

